# Effects of Healthy Ageing on Activation Pattern within the Primary Motor Cortex during Movement and Motor Imagery: An fMRI Study

**DOI:** 10.1371/journal.pone.0088443

**Published:** 2014-06-02

**Authors:** Nikhil Sharma, Jean-Claude Baron

**Affiliations:** 1 Department of Clinical Neurosciences, University of Cambridge, Cambridge, United Kingdom; 2 Institut national de la santé et de la recherche médicale (INSERM), Centre de Psychiatrie et Neurosciences, Hopital Sainte-Anne, Université, Paris, France; Institute of Psychology, Chinese Academy of Sciences, China

## Abstract

The increase in older adults over the coming decades will be accompanied by a greater burden of chronic neurological diseases affecting the motor system. The motor system adapts to maintain motor performance with the primary motor cortex (BA4) emerging as a pivotal node within this neuroplastic process. Studies of ageing often consider BA4 a homogenous area but cytoarchitectonic studies have revealed two subdivisions, an anterior (BA4a) and posterior subdivision (BA4p). Here we focus upon the effects of ageing on the involvement of BA4a and BA4p during movement and motor imagery (MI). Thirty-one right-handed healthy volunteers were recruited and screened for their ability to perform imagery (5 subjects excluded). The sample was split into an older group (n = 13, mean age 56.4 SD 9.4) and a younger group (n = 13, mean age 27.4 SD 5.3). We used an fMRI block-design (auditory-paced [1 Hz] right hand finger-thumb opposition sequence [2,3,4,5, 2…]) with MI & rest and actual movement & rest. We explored the distribution-based clustering and weighted laterality index within BA4a and BA4p. The involvement of BA4p during MI (measured with distribution-based clustering) was significantly greater in the older group (p<0.05) than in the younger group. Hemispheric balance of BA4p decreased with age during MI (Spearman rho −0.371; p<0.05), whereas that of BA4a decreased with age during actual movement (Spearman rho = −0.458 p<0.01). Irrespective of age, we found BA4 is involved during motor imagery, strengthening the rationale for its potential use in older subjects. These findings suggest that the functions of the subdivisions of BA4 are differentially affected by ageing and have implications regarding how ageing affects the cognitive processes underlying motor functions.

## Introduction

The aging of the world population poses a major health challenge. The increase in older adults will be accompanied by a greater burden of chronic neurological diseases many of which affect the motor system. Throughout life the motor system is capable of change, or neuroplasticity. The primary motor cortex (BA4) has emerged as a key area within this plastic process [1]–[5]. Here we will explore how the involvement of the motor cortex alters with aging. More specifically we will compare movement to motor imagery and, for reasons explained below, focus on the two subdivisions of the motor cortex [6].

The motor system constantly adapts to achieve and maintain optimal motor performance [7]. Whether it is changes in anatomy (during growth) or learning a new motor skill, this form of neuroplasticity is critical. Eventually, as a consequence of normal ageing, it must adjust to a motor system that is deteriorating. This decline occurs within both the central and peripheral nervous system and includes reduced levels of neurotransmitters [8], alterations in the white matter [9], and numerous changes to the peripheral nervous system [10]. Functional imaging studies of older subjects suggest that during hand movement there is over-activation of the contralateral motor cortex (BA4) [11], [12]. Yet other studies have reported conflicting results, one study failed to show any age-related increase in the contralateral BA4 activation during a very simple motor task [13] whilst another reported a decrease [14]. Similarly numerous studies suggest that the motor-related network becomes more bilaterally activated with increasing age [11]–[13], with a shift in BA4 hemispheric balance towards the ipsilateral hemisphere [15]. All of these studies rely on active tasks and therefore depend upon motor performance; as such they are complicated by age-related changes that occur distal to the central nervous system.

Motor imagery provides an alternative means to access the motor system, that in principle, is not influenced by more distal age related changes. It is defined as the mental rehearsal of a first person action representation without movement that is confined by the principles of motor control [16]–[18]. It is the close similarity to movement that makes motor imagery such an appealing adjunct. There is close temporal coupling between motor imagery and movement, i.e. the time taken to mentally perform an action closely mirrors the movement [16]. During imagined movement, the reduction in accuracy with increasing speed (i.e. Fitt’s Law) is maintained [18]. The similarities between motor imagery and movement extend to the cortical activation patterns. In healthy volunteers, motor imagery shares many neural substrates with executed movement though some reviews [2], [19] have highlighted important inconsistencies that cannot be attributed to technical differences but more likely secondary methodological flaws.

To date studies of ageing have largely considered the motor cortex a homogenous area but modern cytoarchitectonic studies have revealed two distinct subdivisions, an anterior (BA4a) and posterior subdivision (BA4p) [6]. In addition to distinct cytoarchitecture each subdivision has different receptor densities raising the possibility of differential functions [6]. And yet the relative contribution of its two subdivisions, BA4a and BA4p, to aging is unknown. Key functions attributed to the primary motor cortex may in fact depend upon distinct contributions from one or both of the subdivisions. Functional imaging studies of movement have suggested that BA4p may be more active with aging though the areas were labelled visually.

We have previously reported that motor imagery involves greater activation (assessed with cluster distribution) of BA4p than movement [20]. This led to the suggestion that BA4a is more involved in aspects of movement that discharge via the CST whereas BA4p is involved in more ‘up-stream’ cognitive processes [1], [21]. Here we will focus upon the affects of aging on the involvement of the subdivisions of the motor cortex during movement and motor imagery. In keeping with previous reports, we expect motor imagery to highlight changes that are not apparent during actual movement [20]–[22], and hence to provide novel insights into changes in motor cortex organisation independent of peripheral changes. We hypothesize that motor imagery will involve BA4p (assessed with cluster distribution) more in older subjects compared to young volunteers. In keeping with models of ageing we expect both motor imagery and actual movement to become more bilateral (assessed with hemisphere balance) with age but expect motor imagery will involve BA4p whereas actual movement will involve BA4a. The aim of this study is therefore, by using motor imagery in combination with movement, to examine the effects of normal aging on primary motor cortex neurofunctional organization.

## Methods

### Subjects

31 healthy volunteers were recruited through local advertisement. Mean age was 43.8 yrs (SD 17.1 Range 20–72) 13 were male. Subjects were medically screened and those recruited had no past or present medical history of any neurological, psychiatric or musco-skeletal disorders and were not taking regular medication. Younger subjects included those from our previous report [20]. All subjects were right handed as assessed by the Edinburgh Handedness scale [23].

Ethics Statement: All subjects gave written consent in accordance to the declaration of Helsinki. The protocol was approved by the Cambridge Regional Ethics Committee.

All subjects were assessed using the Chaotic motor imagery assessment battery (see below) and were excluded if unable to perform motor imagery adequately.

### Chaotic Motor Imagery Assessment Battery

Chaotic motor imagery is defined as either an inability to perform motor imagery accurately or temporal uncoupling of otherwise accurate imagery [2]. Subjects were assessed using the Chaotic motor imagery Assessment Battery [2], [21], [22], [24], [25] which has three components that provide an objective measure of motor imagery compliance (see [2], [21], [22] for a full description). The first component used a hand mental rotation task to assess implicit motor imagery: subjects who score below 75% are excluded. The second component used variation of the fMRI task with a variable block length to ensure motor imagery performance. The third component used principles of motor control (more specifically Fitts Law see [2], [21], [22], [24], [25]) to ensure subjects are using motor imagery: subjects using alternative cognitive strategies such as counting are excluded. During all motor imagery tasks, subjects were instructed to perform first-person motor imagery; not to view the scene from the 3rd person; and not to count, assign numbers or tones to each finger. Subjects were excluded if they were unable to perform motor imagery.

### Functional MRI

#### Motor paradigm and fMRI

The fMRI used an established block design [20]–[22] with auditory pacing (1 Hz) of a right hand finger-thumb opposition sequence (2,3,4,5; 2…) with two separate runs (MI & rest and EM & rest). As activation patterns during MI may be unduly influenced if preceded immediately by execution [26], this was performed after MI. Subjects were instructed to keep their eyes closed. We used individually calibrated bilateral fibre-optic gloves (Fifth Dimension Technologies, SA) to monitor finger movements, exclude inappropriate movement and to assess the performance of motor imagery. After each motor imagery block, subjects confirmed whether the finger they were currently imagining was the correct ‘stop finger’ for the length of sequence. After scanning subjects were asked to rate the difficulty of motor imagery performance on a seven point scale [27].

#### Data acquisition

A 3-Tesla Brucker MRI scanner was used to acquire both T2-weighted and proton density anatomical images and T2*-weighted MRI transverse echo-planar images sensitive to the BOLD signal for fMRI (64×64×23; FOV 20×20×115; 23 slices 4 mm, TR = 1.5 s, TE 30 ms, Voxel Size 4×4×4).

#### Image analysis

Each run was pre-processed separately using Statistical Parametric Mapping software (SPM 8). Images were corrected for acquisition time delays, spatially realigned (movement <1 mm), transformed into the standard space of the Montreal Neurological Institute [28] and smoothed (FWHM 6 mm).

As in our previous articles [20], the BA4a and BA4p ROIs used in this study were based upon the published probabilistic cytoarchitectonic maps [29]. Briefly, these maps were generated using ten post mortem brains that underwent a T1-weighted MRI scan prior to observer-independent cytoarchitectonic analysis, the combination of which were subsequently normalised to the MNI single-subject template. The probability of each voxel to belong to each cytoarchitectonic area was then computed. Any voxel that was found to belong to a given region in at least 5/10 of the brains was assigned to that region, which occurred for 91.6% of all voxels. The remaining voxels were assigned to the region with the greatest probability, taking into account the probability of the surrounding voxels. The BA4a and BA4p ROIs derived from the standard cytoarchitectonic *maximum* probability maps (MPMs), i.e. where each voxel is assigned to one cortical area to produce a continuous non-overlapping binary parcellation of BA 4.

#### Distribution-based cluster labelling

To precisely assess the topography of activation clusters within Ba4a and BA4p, we used “distribution-based cluster assignment”, as described in detail by Eickhoff [30] and implemented using the probabilistic cytoarchitectonic Toolbox within SPM [29], [30]. Briefly, this method provides probabilities for clusters and peak voxels to belong to cytoarchitectonic areas as with Eickhoff, et al. 2005, but in addition assesses the distribution of probabilities across voxels to belong to a given area relative to the distribution of probabilities for that particular area, i.e., it takes into account the true unthresholded probabilities rather than the thresholded MPMs [30]. The aim is to assess the “central tendency” of clusters, i.e., differentiating if a cluster is located predominantly toward the centre of or “peripherally” in a region. This is achieved by comparing the overlap of the activation cluster with the un-thresholded probability map for each anatomical area (i.e. containing a full range of probabilities from 10% to 100% for that anatomical area). This is represented by an expression of whether the activation cluster exceeds the expected probability distribution of that area, where the mean probability of the activation cluster (calculated by the average probability across all individual voxels for that anatomical area) is divided by the overall mean probability of the anatomical area, to yield a quotient called *P_excess_*. This quotient represents how much more (or less) likely the anatomical area was present in the activation cluster than would be expected if the probabilities of the anatomical area followed their overall distribution. Hence a *P_excess_*>1 suggests a rather central location of the activation cluster with respect to the anatomical area, whereas a *P_excess_*<1 implies a more peripheral one.

#### First level analysis

Single-subject fixed-effect analysis was performed for each task, and the subsequent con image used in second level analysis. A binary mask of the left BA 4 ROI was applied to each model using a threshold of p<0.001 (Small Volume Corrected). For each subject the cluster distribution within BA 4 was assessed using the method described above to generate a *P_excess_* for both Ba4a and BA4p for each task. A repeated-measures ANOVA was performed using factors (TASK- MI and EM; AREA - BA4a and BA4p) and between GROUP (Age–Old, Young; see Results). Significant effects were further explored using *post-hoc* paired t-tests.

#### Second level analysis

The contrast images [31] from the first level analysis were used for the second level analysis (Random Effect). The contrast image was entered into a full factorial model (Task EM, MI; Age Old, Young). Whole brain contrasts were performed with a p value of 0.05 corrected for multiple comparisons (Family Wise Error-FWE). Voxel-based region of interest analysis was performed in the contralateral BA4 (containing BA4a and BA4p) using a p<0.001 cut-off with Small Volume Correction (SVC).

#### wLI analysis

We then examined the weighted laterality index (wLI) for BA4a (wLI**_BA4a_**) and BA4p (wLI**_BA4p_**). wLIs were derived using a previously described method [15], [32]. The wLI takes into account inter-subject variations in global activation levels and has been shown to be less subject to ceiling and floor effects than the classic LI [15]. The wLI would be −1 in exclusively right hemisphere activations and +1 if exclusively left hemisphere. Due the expected non-normal distribution of the wLI measurement, a non-parametric Mann-Whitney was used to compare wLIs between groups, and Spearman correlations to examine the relationship with Age (1-tailed based upon hypothesis).

## Results

### Behavioural Results

In total 5 subjects were excluded; three because of a failure to perform implicit motor imagery satisfactorily (mean Score 69.9%); two because of the use of alternative cognitive strategies (mean break point +23%). The remaining 26 subjects (12 male) performed adequately on all aspects of the hand rotation task (mean 94.4% SD 4.3%) fMRI simulation and Fitts law (mean break point 17.4% less for motor imagery then movement) as well as during the MRI session. The sample was split in half into two subsets of equal size, an older group (Age>40, n = 13, mean age 56.4 SD 9.3) and a younger group (Age<40, n = 13 mean 27.4, SD 5.3). Median post MRI motor imagery scores (MIS) was, Right hand = 6 and there was no difference between the groups. No subject failed to either suppress movement or showed evidence of non-compliance during the fMRI paradigm.

### fMRI Data: 1^st^ Level Distribution-based Cluster Labelling

The repeated-measures ANOVA demonstrated an effect of AREA on *P_excess_* [F(1, 24) = 51.744, p<0.001], with no significant effect of TASK [F(1, 24) = 0.062, p<0.805]. There was a significant interaction between TASK and AREA [F(1, 24) = 6.51, p<0.05] and between AREA and GROUP [F(1, 24) = 6.70, p<0.05].

**Figure 1:**
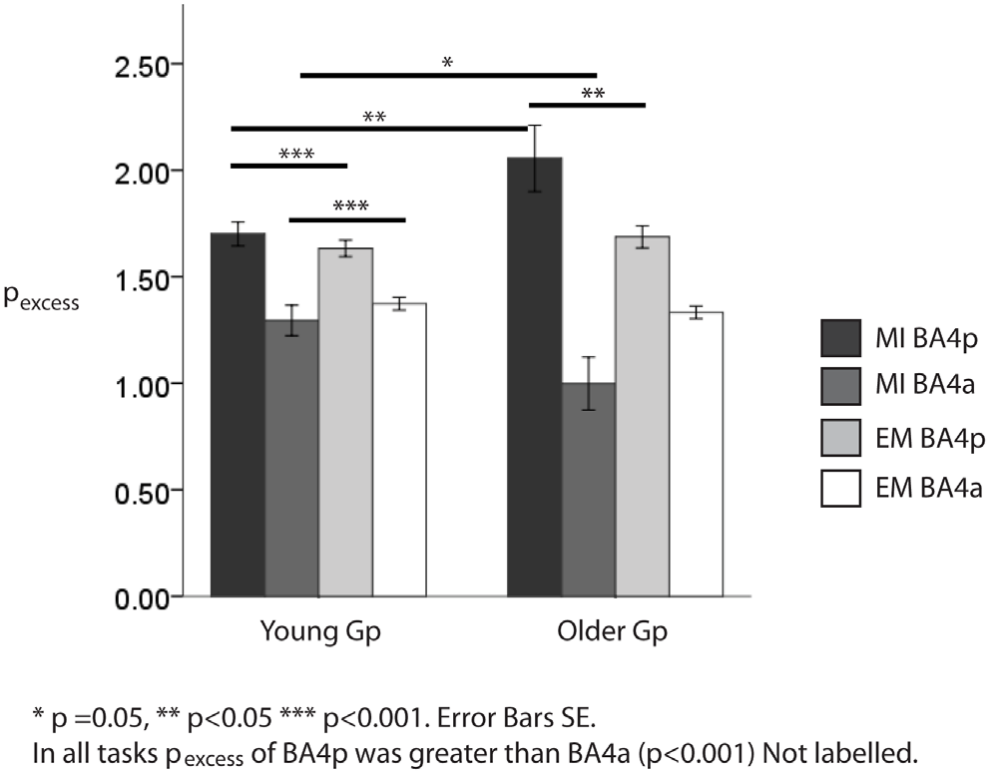
The cluster based distribution for each task in both groups.

Further differences were explored with post-hoc t-tests (see **Figure 1**). A consistent finding across groups and tasks was that BA4p *P_excess_* was significantly greater than *P_excess_* BA4a.

With regards to BA4p *P_excess_* it was significantly greater during motor imagery than actual movement in both groups (p<0.001 and <0.05 for the young and older group, respectively) and it was significantly greater in the older than the younger group (p<0.05) during motor imagery.

With regards to BA4a *P_excess_*, it was significantly greater during actual movement than during motor imagery in the young group only (p<0.001) and it was significantly greater the younger group than the older group (p = 0.05).

In summary, motor imagery revealed age-related changes in *P_excess_* that were not apparent during actual movement. Particularly, *P_excess_* BA4p was significantly greater in the older group, whereas *P_excess_* BA4a was significantly lower.

### fMRI Data: 2^nd^ Level Analysis

**Figure 2:**
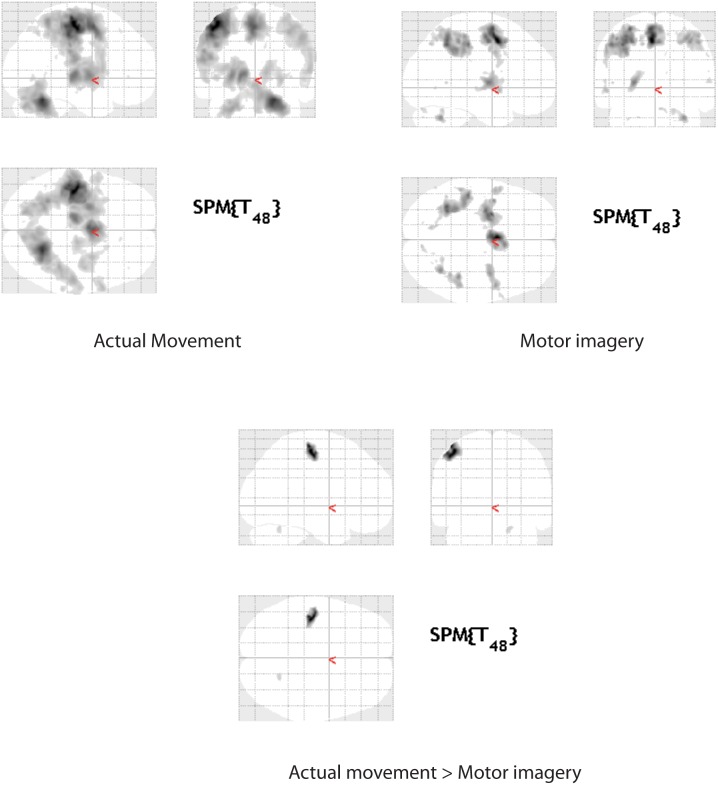
Whole brain activations for actual movement, motor imagery and actual movement > motor imagery (FWE P<0.05).

Using whole brain analysis across the whole group (n = 26), the regions activated are shown in Table 1 & Figure 2, and the direct comparison between motor imagery and movement in Table 2 & Figure 2. Overall the activations for both tasks are in keeping with those we have previously reported, involving mainly contralateral BA4, PMd, parietal lobe and ipsilateral cerebellum for both movement and motor imagery, although the involvement of BA4 during Imagery was significantly less, and more caudal to the hand area, than with executed movement.

**Table 1:** Whole Brain Analysis: Actual movement & motor imagery.

*Task*	*Cluster*	*T-value*	*Coordinates*				*Location*	*Task*	*Cluster*	*T-Value*	*Coordinates*				*Location*
			x	y	z						x	y	z		
***Executed***	43817	16.66	−42	−20	58	**L**	**BA6/BA4**	***Motor***	6011	9.63	−3	6	52	**L**	**SMA**
***Movement***		13.64	−48	−26	47	L	S1	***Imagery***		8.46	−4	0	64	L	SMA
		11	−32	−9	57	L	BA6			7.45	4	14	50	R	SMA
	28925	14.31	21	−55	−24	**R**	**CB**		4419	8.2	−28	−1	63	**L**	**BA6/BA4**
		9.27	−25	−53	−32	L	CB			7.95	−32	−7	55	L	BA6
		8.98	8	−64	−18	R	CB			7.33	−42	2	53	L	MFG
	12971	12.9	−1	2	55	**L**	**SMA**		9210	8.13	−36	−50	46	**L**	**hIP1**
		6.63	−4	−18	52	L	BA6			7.75	−48	−27	46	**L**	**S1**
	23246	11.54	−13	−19	5	R	Thal-pref			7.48	−34	−40	41	L	hIP3
		10.63	−25	−3	3	**L**	**Thal-M**		2542	7.66	−23	−3	6	**L**	**Putamen**
		8.62	−25	−20	7	L	Putamen			6.18	−26	5	−1	L	Putamen
	14023	8.81	39	−36	50	R	S1			5.84	−29	−17	−1	L	Putamen
		8.74	53	−21	44	**R**	**S1**		851	7.28	33	−57	−32	**R**	**CB**
		7.74	40	−49	48	R	hIP2			6.06	21	−55	−24	R	CB
	4027	8.2	13	−16	7	**R**	**Thal-pref**		2108	7.22	40	−38	51	R	S1
		6.47	13	−1	12	R	Thal			6.73	48	−36	56	**R**	**S1**
		6.38	10	−9	−8	**R**	**Thal**			6.57	38	−47	52	R	SPL (7PC)
	3255	7.98	61	12	9	**R**	**BA44**		2677	7.04	36	−2	54	R	MFG
		7.38	63	11	22	**R**	**BA44**			6.91	49	4	49	**R**	**BA6**
		6.67	55	3	−1	R	BA44			5.98	28	−5	57	R	SFG
	3773	7.98	40	−9	57	**R**	**BA6**		712	6.58	−54	4	39	**L**	**BA6**
		7.58	34	−11	63	**R**	**BA6**			5.87	−54	7	25	**L**	**BA44**
		6.69	35	−1	66	R	SFG		338	6.43	−54	8	−2	L	Temp
	2165	7.88	29	0	−2	**R**	Putamen			5.86	−55	11	7	L	BA44
		5.88	18	16	6	R	Caudate		209	6.42	−41	−2	42	**L**	**BA6**
	328	7	−43	8	1	**L**	**Insula**		276	6.33	−13	−68	47	L	SPL
	412	6.84	14	−60	48	**R**	**Precun**			6.15	−15	−62	53	**L**	**SPL7A**
		6.05	15	−70	54	**R**	**SPL7P**		138	6.19	7	−78	−22	**R**	**CB**

**Table 2:** Whole Brain Analysis: Direct Comparison between conditions.

*Task*	*Cluster*	*T-Value*	*Coordinates*			*Location*
			x	y	z	
Actual movement > Motor imagery	2283	13.73	−43	−23	63	L PMd
		8.78	−46	−17	54	L PMd
		8.59	−44	−18	55	L Ba4a
	214	5.86	21	−55	−23	R CB
Motor imagery > Actual movement	Nil	n/a	n/a	n/a	n/a	n/a

During executed movement there was a cluster of voxels that were significantly increased in the older group compared to the younger group within the contralateral BA4 ROI (p<0.001 SVC corrected, 19 voxels t-value 3.4. Talairach coordinates x = −46 y = −9 z = 43), localised to BA4a. There were no other significant activations or interactions.

### Weighted Laterality Index

As expected, wLI was not normally distributed (Kolmogorov-Smirnov p<0.001). There was no significant difference between young and old groups for wLI_BA4a_ (Young group wLI_BA4a_ MI 0.48, EM 0.71; and Older group wLI_BA4a_ MI 0.47, EM 0.68;). There was a trend for wLI_BA4p_ to be low in the older group Mann-Whitney p = 0.064 (Young group wLI_BA4p_ MI 0.77, EM 0.87 and Older group wLI_BA4p_ MI 0.62, EM 0.89).

**Figure 3:**
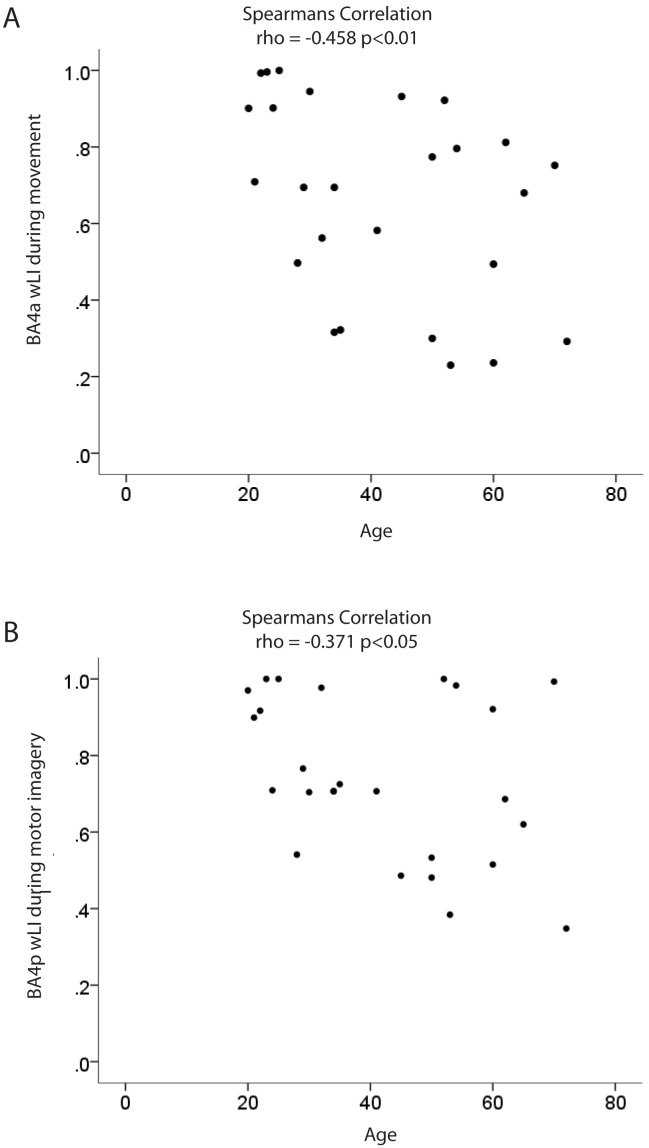
Spearman correlations of Age and (A) wLI BA4a and (B) wLI BA4p.

During actual movement there was a significant decrease in wLI_BA4a_ with age (see Figure 3**A**; Spearman rho = −0.458 p<0.01), i.e. the hemispheric balance of BA4a become more bilateral with increasing age. During motor imagery the wLI_BA4p_ decreased with age (see Figure 3**B**; Spearman rho = −0.371; p<0.05), i.e. the hemispheric balance of BA4p becomes more bilateral with increasing age.

The resulting shift in wLI was a consequence of increased activation of the ipsilateral BA4a during actual movement (Spearman rho = 0.388 p<0.05) and ipsilateral BA4p during motor imagery (Spearman rho = 0.335 p<0.05).

## Discussion

In this study we demonstrate that irrespective of age, BA4 is involved during motor imagery strengthening the rationale for it’s use in aged subjects. In keeping with our previous report in a subset of the present sample [20], the cluster distribution of BA4p (measured with *P_excess_*) was greater during motor imagery than executed movement. However, the present finding that the cluster distribution of the BA4p during motor imagery was greater in the older group than in young volunteers is new. We also document for the first time that during motor imagery the hemispheric balance of the *posterior* subdivision of BA4 (BA4p) decreases with age, while during executed movement it is the hemispheric balance of the *anterior* subdivision of BA4 (BA4a) that was found to decrease with age. Overall these results indicate that the functions of the subdivisions of BA4 contralateral to executed or imagined movement are affected by aging, and that the hemispheric balance of the BA4 subdivisions is also differentially affected by aging.

This study demonstrated that appropriate motor imagery screening is important in older subjects. We found that nearly 24% of subjects over 40 yrs old were excluded due chaotic motor imagery. Not only is this relevant to functional imaging studies (with small recruitment numbers) but also to the investigation of motor imagery training where inclusion of such subject may dilute any beneficial effect. Had we included these subjects our results may have been different; given the excluded subjects were not performing motor imagery they will have likely added noise to the data. Our findings are keeping with reports that older subjects have a reduced capacity to perform motor imagery [33]–[37]. It is unknown what impact the reduction in capacity to perform imagery has on activities of daily living for healthy volunteers.

While we have focused on the subdivision of the motor cortex we also report whole brain activation result. There are few studies that have explored the cortical activation patterns in elderly subjects using motor imagery [38]–[40]. Nedelko et al [40] focused on the mirror neuron system but found no differences in older subjects during motor imagery. Zapparoli et al [38] reported greater activation of occipito-temporo-parietal areas in elderly subjects versus young subjects. There are a number of reasons that explain why these findings were not replicated here. The first is that Zapparoli et al used conjunctional analysis of their imaging data which can be driven by outliers. Secondly and as the authors admit, it is likely that their subjects were depending of visual imagery rather than motor imagery. The chaotic motor imagery assessment will have excluded subjects who use this strategy in our study.

In this study we have focused on the motor cortex and more specifically its two subdivisions. We have done so given BA4’s importance in motor learning [1], [6], [41], the recovery process [3], [42] and its ongoing use in brain computer interfaces [43]. The two subdivisions of BA4 have been overlooked for many years [6]. Human ex-vivo studies report that each subdivision has distinct receptor densities and cytoarchitecture [6] suggesting that BA4a and BA4p have distinct functions [1]. It is likely that characteristics previously attributed to BA4 may in fact be a function of each or both subdivisions. BA4p has direct connections to spinal motoneurons in the ventral horn (cortico-motoneuronal (CM) connections [44]), whereas BA4a acts via the spinal interneurons in the intermediate zone [44]. This implies that BA4a and BA4p are likely to be differentially affected by ageing. It has been demonstrated that somatotopically organised areas, for example the thumb, are dually represented within these 2 sub-regions, each with a specific though not exclusive function [6]. We have previously suggested that BA4p is involved in the ‘non-executive’ aspects of motor control. BA4a is thought to be more ‘executive’ in nature, i.e. resulting in actual movement, whereas BA4p appears to be involved in a number of cognitive tasks or non-executive functions. Initially BA4p was shown to be activated by sensory inputs [6], but it has subsequently been shown to be modulated by attention [45], [46] and affected by normal ageing [12].

With regards to executed movement, our results from the voxel-based analysis suggest the degree of age-related differences in the contralateral BA4 is consistent, with earlier studies [11], [12], [15], [47]. The task complexity may influence BA4 activation [48] with tasks requiring greater coordination producing greater age-related increases while rate of pacing appears to be less important [47]. This may partly explain why studies that used simple motor tasks failed to find any age-related changes in BA4 activation [13], [49]. We report that the age related cluster during executed movement localises to BA4a. Ward et al reported non-linear aged-related changes in BA4p, a difference with our findings that could relate to the difference in fMRI paradigm; a finger tapping exercise here as compared to a grasping task with visual feedback. Furthermore in Ward et al the degree of smoothing was much greater and BA4p was identified visually rather than using probabilistic cytoarchitectonic maps.

In keeping with previous reports motor imagery has highlighted changes that are not revealed during executed movement [20]–[22]. We found that the cluster distribution (i.e., *P_excess_*) of BA4p was greater in the older group during motor imagery than the younger group. BA4p has been shown to be modulated by attention and it is conceivable that older subjects attended to the task more, however if this were the case then one would expect the cluster distribution to relate to the motor imagery score (a subjective scale of how difficult the task was) which it does not. The explanation is likely to involve the similarities but also the differences between these tasks. For instance, it is plausible that the age-related changes for BA4p during imagery result from a high-order process such as planning, whereas the changes involving BA4a during executed movement related to the executive process itself. Whether this is a result of peripheral degradation of perhaps the CST or peripheral motor system requiring greater executive drive is possible but requires further investigation. Of note TMS evoked corticomotor facilitation by motor imagery is largely preserved in the older subjects [50], [51]. To date however motor evoked potentials have not been considered in the framework of the two subdivisions of BA4, and the role of each subdivision in the motor evoked potential is unknown. We could speculate that as TMS evoked corticomotor facilitation by motor imagery is preserved in ageing BA4p may make a significantly contribution.

We have demonstrated that, mainly reflecting increases in BA4 activation, the degree of hemispheric BA4 activation laterality decreases with age for movement and motor imagery. However that effect involved BA4p with motor imagery, and BA4a with executed movement. This difference in effect of aging on BA4 subdivision hemispheric balance is likely to represent a difference in the cognitive aspects of each task, as discussed above. Nevertheless the overall shift to a more bilateral appearance is consistent with models of aging such as the Hemispheric Asymmetry Reduction in Older Adults (HAROLD) [52] that suggest that in order to maintain performance during aging hemispheric lateralisation decreases. Specifically with regards to the motor system, we confirm here our previously reported shift in BA4 hemispheric balance during executed movement [15]. A key novel finding from the present study is that during executed movement this shift in hemispheric balance lay within the anterior subdivision (BA4a), and within the posterior subdivision during motor imagery.

Overall the findings from the present study reinforce the notion that motor imagery is integral to the motor system but suggest that the functions of the subdivisions of BA4 are differentially affected by ageing. To date the functions of BA4a and BA4p are largely unknown. Understanding the functions and interdependence of BA4a and BA4p is likely to lead to a richer understanding of the ageing process. In turn this will strengthen our understanding of how best to exploit the neuroplastic process to reduce disability in chronic neurological diseases such as stroke.
